# A Comprehensive Review of the Current Research Status of Biodegradable Zinc Alloys and Composites for Biomedical Applications

**DOI:** 10.3390/ma16134797

**Published:** 2023-07-03

**Authors:** Lingyun Kong, Zahra Heydari, Ghadeer Hazim Lami, Abbas Saberi, Madalina Simona Baltatu, Petrica Vizureanu

**Affiliations:** 1 School of Electronic Information, Xijing University, Xi’an 710123, China; klyindhtm@126.com; 2 School of Electrical and Computer Engineering, College of Engineering, University of Tehran, Tehran 1439957131, Iran; heydarizahra372@gmail.com; 3 Department of Material Engineering, South Tehran Branch, Islamic Azad University, Tehran 1777613651, Iran; engineeras75@gmail.com; 4 Department of Biomedical Engineering, Islamic Azad University, South Tehran Branch, Tehran 1777613651, Iran; 5 Department of Technologies and Equipments for Materials Processing, Faculty of Materials Science and Engineering, Gheorghe Asachi Technical University of Iaşi, Blvd. Mangeron, No. 51, 700050 Iasi, Romania; cercel.msimona@yahoo.com

**Keywords:** Zn-based alloys and composites, mechanical properties, biological properties, biomedical applications

## Abstract

Zinc (Zn)-based biodegradable materials show moderate degradation rates in comparison with other biodegradable materials (Fe and Mg). Biocompatibility and non-toxicity also make them a viable option for implant applications. Furthermore, Pure Zn has poor mechanical behavior, with a tensile strength of around 100–150 MPa and an elongation of 0.3–2%, which is far from reaching the strength required as an orthopedic implant material (tensile strength is more than 300 MPa, elongation more than 15%). Alloy and composite fabrication have proven to be excellent ways to improve the mechanical performance of Zn. Therefore, their alloys and composites have emerged as an innovative category of biodegradable materials. This paper summarizes the most important recent research results on the mechanical and biological characteristics of biodegradable Zn-based implants for orthopedic applications and the most commonly added components in Zn alloys and composites.

## 1. Introduction

**Figure 1:**
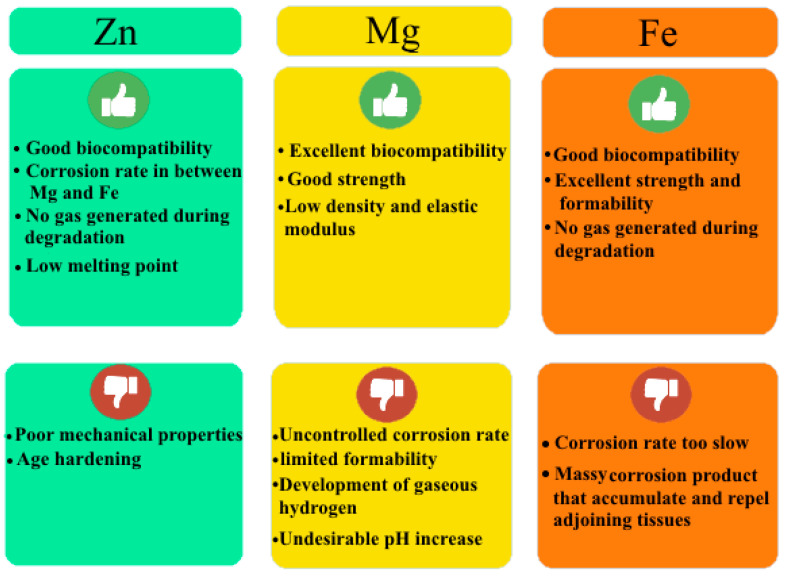
Strengths and weaknesses of Mg-, Fe-, and Zn-based BMs.

The World Health Organization (WHO) ranks osteoporosis as the second most dangerous condition in human health. The risks of senile osteoporosis are growing more hazardous as the aging process in human society accelerates, and the danger of osteoporosis-caused bone fractures in people is rising. Furthermore, osteoporosis seems to be affecting people at earlier ages. Fracture fixation implants currently primarily contain non-biodegradable metals such as stainless steels and cobalt (Co) and titanium (Ti) alloys, which either perpetually endure in the body or require surgical follow-up to eliminate them after fracture curing. As a result, using bioabsorbable metal elements to heal bone fractures is extremely desirable [1,2,3]. The characteristics of natural body bones, for comparison with non-biodegradable and biodegradable metals (BMs), are given in Table 1. BMs are those that will increasingly corrode within the organic structure and disappear, whereas the aim is to care for tissue regeneration without implant residuals. The released corrosion product ought to cause the lowest toxic response; therefore, the primary design should evade victimization components [4,5]. It is known that an ideal implant must not only have gradual bone loss at an appropriate rate (about 0.2–0.5 mm.year^−1^) to suit the healing process, but also have biological activity (the ability to directly bond natural bone) to facilitate the bone healing process [6,7,8,9,10,11,12,13,14]. On the other hand, the elastic modulus should be as close to the cortical as possible to avoid stress shielding (10–20 GPa). The yield strength (YS), ultimate tensile strength (UTS), and elongation to failure should be above 230, 300 MPa, and 15–18%, respectively. In addition, mechanical integrity must be maintained for more than 6 months [15,16,17,18,19]. Prime absorbable metals embrace iron (Fe), magnesium (Mg), and Zn [20]. Given that both Mg and Fe cause concerns, the scientific community has continued to explore new solutions [19,20,21,22,23]. Zn and its alloys appear to overcome some of the basic technical challenges related to the usage of biodegradable Mg and Fe. The rewards and disadvantages of these BMs, Mg, Fe, and Zn, are summarized in Figure 1 [18,24,25,26,27,28,29,30]. Mg-based materials show a high rate of degradation, with large amounts of hydrogen released in the human body, which can lead to the disruption of tissue healing; in contrast, Fe has a relatively slow degradation rate in the body and, more importantly, some degradation products may remain at the implantation site. On the other hand, Zn demonstrates reasonable degradation rates, which has attracted increasing interest in terms of replacing Zn alloys with other biodegradable metals [31,32]. In a typical 70 kg human, Zn is the sixth most abundant metal (2.3 g) after Ca (1 kg), K (140 g), Na (100 g), Mg (19 g), and Fe (4.2 g). It occurs more frequently than Rb (0.68 g), Sr (0.32 g), and other trace metals [33,34]. It is no surprise that Ca tops the list—in fact, it is found in amounts of around 1 kg and in 99% of human Ca, and along with phosphate, makes up the skeleton. In addition, Zn is a fundamental mineral that is required for ordinary skeletal development and bone homeostasis. Furthermore, Zn has been shown to be able to promote bone recovery. Be that as it may, the cellular and molecular pathways through which Zn promotes bone development, homeostasis, and regeneration are poorly understood. Zn can emphatically influence chondrocyte and osteoblast capacities, whereas it hinders osteoclast action, consistent with a beneficial role for Zn in bone homeostasis and regeneration. In the twentieth century, researchers developed Zn alloys as a main feature in the rapid production of tools, transmission components, and molds due to their mechanical properties, low melting points, economic advantages, fluidity, processing, and outstanding ductility. Therefore, they compete with other non-ferrous and ferrous alloys [35]. After Fe, Al, and Cu, Zn is the fourth most abundant metal on Earth. Zn is also recycled in significant proportions, with secondary Zn production accounting for 20–40% of world consumption [36]. However, practically all Zn die-casting alloys are made through primary Zn production due to rigorous impurity limits in die-casting composition requirements. In general, nearly half of the Zn usage is consumed to galvanize steel in order to protect it against corrosion [37]. Other notable usages include the use of Zn as an alloy element in brasses, bronzes, Al, and Mg alloys, as well as other coatings. Zn oxide (ZnO) has been applied as an additive in chemical, pharmaceutical, cosmetics, paint, rubber, and agricultural applications [38]. Zn has been called the ‘calcium’ of the twenty-first century because of its many important biological roles in the body, including nucleic acid metabolism, the stimulation of new bone formation, signal transduction, the preservation of bone mass, the regulation of apoptosis, and gene expression. Zn not only prevents bone loss and inflammation-related disease, but also plays an important role in cartilage substrate metabolism (SOX9) and cartilage gene expression II. Thus, compared with Mg and Fe BMs, Zn-based BMs have emerged as the next generation of BMs for bone tissue engineering [39].

**Figure 2:**
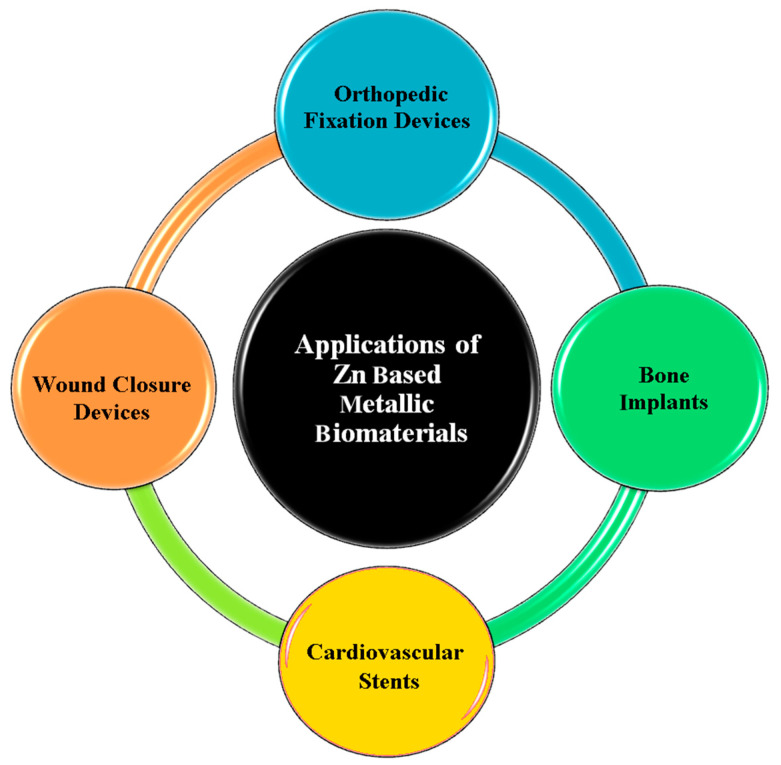
Diverse applications of Zn-based alloys in medicine.

The use of Zn alloys and composites is preferred because their mechanical properties show a significant improvement compared to pure Zn, as well as alloying or composites, if carried out correctly and can provide multiple benefits for various applications, such as in bioresorbable implants. Previous research has shown that, due to the high cellular compatibility of various Zn alloys including Zn-Mg, Zn-Ca, Zn-Mn, Zn-Li, Zn-Sr, etc., there is a tendency to use them as biodegradable implants. It has also been shown that the addition of bioceramic particles and carbon elements can significantly improve elongation and UTS and other mechanical properties parameters [49,50,51]. The various applications of Zn and its composites in medicine are shown in Figure 2. To date, there have been few review papers specializing in the mechanical behavior of various Zn-based alloys and composites in orthopedic applications. During this review, the influence of diverse elements on Zn and its alloys, as well as providing reinforcements to Zn composites, the mechanics and biocompatibility performance are comprehensively assessed. We also summarize the present understanding of the possible mechanical mechanisms of Zn and its composites and fabrication methods. Furthermore, the key challenges are investigated, with attention paid to research on Zn-based implants.

## 2. Zn Alloy Candidates for Biomedical Applications

Zn, a BM, possesses extremely good biodegradability and biocompatibility, but its low mechanical specifications have limited its biomedical applications. The addition of alloying elements could typically be recommended as an efficient technique for enhancing the mechanical features. As cardiovascular and orthopedic implants require high hardness and strength, the development of Zn mechanical characteristics is one of the most important approaches by which to expand its application in medical implants [49,50,51,52]. Alloying elements are used as a good tool to significantly improve the performance of Zn [53,54,55,56,57]. A number of the mechanical characteristics of Zn-based alloys are compared in Table 2.

### 2.1. Pure Zn

Zn plays an important role in human health as an enzymatic element because it is the main component of the protein structure, as well as a transcriptional regulator of biochemical and cellular processes. Genes associated with Zn proteins make up approximately 3% of the human genome, highlighting the significance of Zn in numerous functions. The many effects of Zn are in bone creation/resorption as a consequence of the stimulating effect of Zn on bone formation and mineralization in osteoblasts and the stimulation of protein synthesis. Nutritional Zn also prevents bone loss resulting from bone disorders. Zn and its alloys have recently been manufactured as orthopedic implants, as the results show the likelihood of pure Zn to cause new bone formation and osseointegration two months post operation [72,73]. Recently, thanks to its properties, Zn has been considered a bio-absorbable element. Pure Zn has been extensively researched as an implant to strengthen bones. It is the most significant essential element within the body and an individual consumes approximately 40 mg/day. The studies have also shown the importance of Zn in cell proliferation, differentiation, and the arrangement of the extracellular matrix, as well as in bone growth and healing [47]. Zn deficiency can cause hypothermia, dystocia, hypotension, hair loss, bleeding, neuropathy, failure to thrive, dermatitis, and diarrhea [74]. However, it is important to note that Zn poisoning is a medical condition associated with an overdose or excessive exposure to Zn. Such levels of toxicity have been observed with the ingestion of more than 50 mg of Zn. Following ingestion of extremely high doses of Zn (of which 300 mg of Zn/day—20 times the US RDA—is a “low intake” overdose), pain, vomiting, nausea, and cramps may occur [75]. Zn also demonstrates relatively low corrosion rates, and Zn demonstrates the property of not converting to hydrogen during its decomposition, thus avoiding the harmful effects resulting from the accumulation of hydrogen within the surrounding tissues, which avoids causing negative effects on the bone healing process. It is worth mentioning that the UTS of Zn is from 100 to 150 MPa and the Young’s modulus is small; therefore, Zn is close to the properties of natural bone. However, the high strength of Zn is smaller than the specified values for implants used for bone healing [47] and its mechanical strength is insufficient for many clinical usages [76].

### 2.2. Zn-Mg Alloys

Zn-Mg alloys are the most widely employed in medical applications because adding Mg to Zn has been shown to significantly enhance corrosion resistance and mechanical properties [72,77]. Pachla et al. [58] investigated the mechanical characteristics of Zn-Mg binary alloys with Mg amounts of 0.5, 1.0, and 1.5% as possible materials to be applied in orthopedic implants. The authors, after casting the material in a rod-shaped mold and extruding it at 250 °C, then re-extruded it using the hydrostatic (HE) method at an ambient temperature. After the HE process, the microstructure of the alloys was constructed from tiny grains (~1 μm) of αZn in a two-phase coat of 50–200 nm nano grains of the αZn + Mg2Zn11 eutectic. A three- to four-fold drop in grain size with HE increased the YS from 100% to over 200%, and the elongation at break from 100% to 30 fold (except for Zn-0.5 Mg alloy), which also had a greater than 50% hardness (except for Zn-1.5 Mg alloys), compared with the results reported in the literature for similar alloys. For the Zn-0.5 Mg and Zn-1Mg alloys, after immersion tests, no corrosion of plasticity was detected. The obtained results were due to major plastic deformations at a high-pressure room temperature using the cumulative HE processes.

Vojtěch et al. [66] produced Zn-Mg alloys with up to 3% Mg and investigated them as possible BMs for medical applications. They compared the microstructure and mechanical and corrosion features of these alloys with pure Mg and Zn-Al-Cu cast alloys. SEM was employed to examine the surfaces of the corroded alloys, and the corrosion behavior of the Zn alloys was used to predict possible Zn dosages and toxicity. These doses were found to be insignificant in comparison to the acceptable biological daily limit for Zn. The Mg concentration and the mechanical properties had a specific relation. They showed that the hardness of Zn-Mg alloys rises with Mg content, ranging from around 25 HV for Zn to 200 HV for ZnMg_3_. This effect can be explained by Mg increasing the amount of the brittle Mg_2_Zn_11_ intermetallic phase. The UTS of Zn-Mg alloys has a somewhat distinct behavior, increasing up to around 1% Mg and then decreasing at greater Mg amounts. The strength of the completely eutectic ZnMg_3_ alloy is equivalent to that of Zn. The large volume proportion of the brittle eutectic in the ZnMg_3_ alloy, as well as the coarse-grained structure of components, contribute to the poor strength. Fracture crack development resistance is minimal in both cases. The resistance to fracture rises in the ZnMg1 and ZnMg1.5 alloys owing to the existence of fine, prime Zn dendrites and a eutectic network. The fine grains, as well as the rigid network, act as obstacles to the developing crack. The optimal volume fractions for structural components related to the ZnMg_1_ alloy have a maximum UTS of nearly 150 MPa. Because the brittle eutectic mixture fills a greater volume in the alloy with higher Mg concentrations, fracturing occurs more rapidly. As a result, YS is only measured for the ZnMg1 alloy, as the other alloys fracture before the plastic deformation is complete [66].

**Figure 3:**
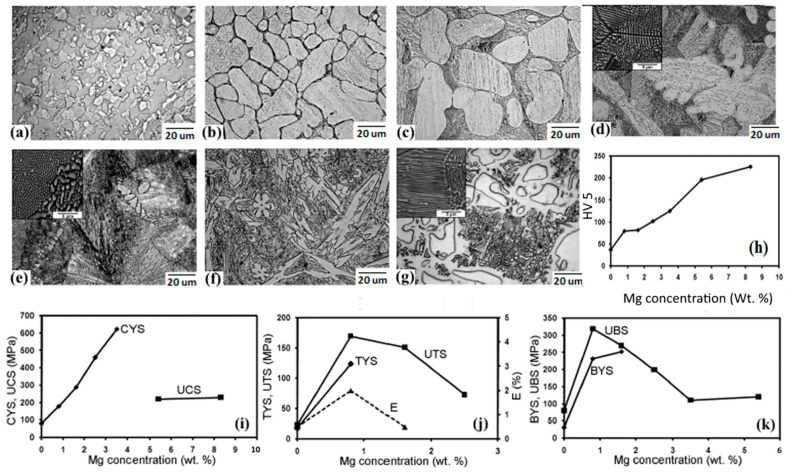
Optical micrographs (with SEM image insets) and mechanical characteristics of: (**a**) pure Zn, and its alloys with (**b**) 0.8 Mg, (**c**) 1.6 Mg, (**d**) 2.5 Mg, (**e**) 3.5 Mg, (**f**) 5.4 Mg, (**g**) 8.3 Mg, (**h**) variations in hardness, (**i**) compressive YS of Zn-Mg alloys regarding Mg amounts, (**j**) variations in tensile characteristics of Zn-Mg alloys with regarding Mg contents, (**k**) bending tests (UBS—ultimate bending strength, BYS—bending yield strength). (Reproduced with permission from Ref. [78]).

Kubasek’s group studied biodegradable Zn-Mg alloys comprising a 0–8.3 weight ratio percentage of Mg, and the microstructural and mechanical features attained were offered phases of Mg_2_Zn_11_ and a eutectic mixture of α-Zn + Mg_2_Zn_11_ [62]. The volume fraction (VF) of the brittle intermetallic compound phase Mg_2_Zn_11_ was observed to increase with incrementing Mg amounts into the Zn matrices. As revealed in Figure 3h,i, the existence of hard intermetallic particles (Mg_2_Zn_11_) strikingly improves the resistance of the Zn matrices under compression, and adding 0.8 wt% of Mg into the Zn matrix improves the σ_UTS_ to 170 MPa; a total 465% growth compared to pure Zn (30 MPa). However, according to the results shown in Figure 3j, high Mg contents in Zn (>0.8 wt%) reduce the tensile features of these alloys. The results are presented in Figure 3k and demonstrate that the plasticity of the Zn-Mg alloy is lower upon bending than during compressive loading. In compression tests, Zn-3.5Mg alloys can bear large plastic deformations, while in bending tests, the alloy is macroscopically brittle due to the difference in the nature of the local stress of the alloy under load. For macroscopic compressive loads, the compressive component of local stress is dominant. Bending, on the other hand, causes local tensile stresses, promoting the creation and growth of macroscopic fracture defects [78]. 

### 2.3. Zn-Ca\Sr Alloys

Pure Zn has a poor strength and fluidity, which is one of its disadvantages as a possible BM. H. Li et al., [46] used three significant IIA critical nutritional elements, Mg, Ca, and Sr, as well as hot-rolling and hot-extrusion thermal deformations, to control this disadvantage of Zn and improve the biocompatibility of Zn alloys. Elements were used to produce the Zn-1Mg-1Ca, Zn-1Mg-1Sr, and Zn-1Ca-1Sr alloys, and played an important role in bone production and influenced the mineral density and strength. The mechanical characteristics of the Zn-1Mg-1Ca, Zn-1Mg-1Sr, and Zn-1Ca-1Sr alloys were substantially greater than those of Zn, as a result of alloying and thermal deformation. In vitro hemolytic rate and cell viability tests revealed that introducing Mg, Ca, and Sr to Zn improves its hemocompatibility and cytocompatibility, ensuring the biosafety of these innovative biodegradable implants in future clinical applications. Furthermore, thermal deformations such as extrusion, forging, and rolling can provide good mechanical characteristics, as can specific heat treatments such as solution treatment and aging [46]. 

### 2.4. Zn-Al Alloys

In industrial Zn-based alloys, Al is the most frequent alloying ingredient. Zn has a solubility of around 1.14 %wt. at 381 °C and about 0.7 %wt. at 277 °C [35]. Al is one of the elements ingested in amounts of 1–10 mg/day from natural sources. Most unprocessed foods comprise small amounts of Al; less than 5 mg/g. Drinking thirty sodas and beers from Al cans and cooking in Al containers statistically increases Al intake due to stent degradation. Due to its potential influences on the reproductive and nervous systems, the suggested intake of aluminum is 10 mg per day for an average-weight person. The biodegradation of a Zn-Al stent with a weight of 50 mg and 5 wt% aluminum is approximately 0.003 mg per day, assuming that the entire stent is bio-resorbed in 2 years and the corrosion rate is stable. For this reason, Al has been a common alloying element in many biomedical implant materials for decades.

Zn-Al is a popular alloy for low-cost die casting, sand casting, rolled strip and foil, and steel hot dip galvanization. Zn-Al alloys are by far the most well-documented Zn alloy family. They are normally classed as hypoeutectic die casting alloys with a content of 5 wt% Al, or hypereutectic foundry alloys with a content of 8 wt% Al [79,80,81]. 

**Figure 4:**
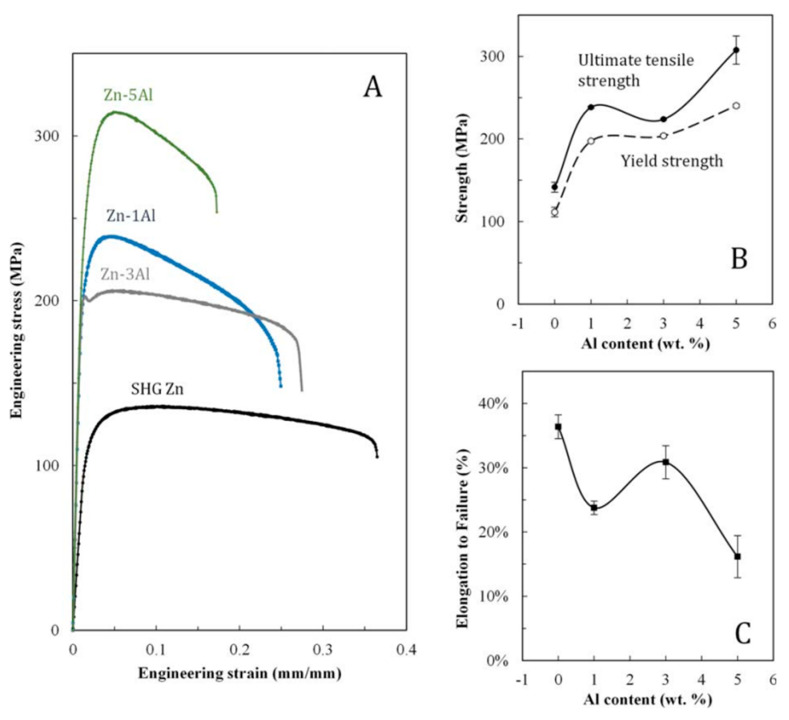
Tensile test results for Zn-Al alloys. Stress-strain curves are presented for the outcomes of one representative specimen (**A**). Average tensile and YSs (**B**) and elongations to failure (**C**) are presented for multiple specimens, with error bars that correspond to plain error. (Reproduced with permission from Ref. [82]. Copyright © 2017 Wiley).

Several studies on biomedical materials have suggested that the presence of Al is associated with an increased risk of Alzheimer’s disease. However, no strong scientific evidence has yet been provided for this theory. Bowen et al. [82] assessed the mechanical properties of Zn-Al; the tensile stress-strain curves in Figure 4 show that an Al content of up to 5.5 wt% enhanced the strength of Zn-Al alloys. An increase in strength did not uniformly occur as the amount of Al rose, with the tensile strength significantly dropping at 3 wt% Al compared with 1 and 5 wt% Al. The UTS showed a significant drop, while the YS did not (Figure 4).

With increased Al content, the elongation to failure was reduced. When the stress-strain curves for alloy Zn-3Al were taken into consideration, a sharp drop in stress was immediately found past the YS. Zn-5Al had the greatest UTS 308 ± 68 MPa and YS 240 ± 7 MPa, and Zn-3Al had the highest elongation at failure (31 ± 3%), which was similar to SHG Zn (36 ± 2%). The strength of all alloys was found to be greater than that of pure Zn [82].

### 2.5. Zn-Li Alloys

Lithium (Li) is commonly utilized in Zn alloys. Thanks to the considerable solubility of Li in Zn, Zn-Li is one of the few possible age-hardenable systems. In vivo investigations of LAE442 (4 %wt Li) implant material have demonstrated its appropriateness for orthopedic applications. In the early 1970s, the U.S. Food and Drug Administration (FDA) authorized Li as a drug to treat manic depression. Brain damage, stroke, Alzheimer’s, Huntington’s, and Parkinson’s illnesses, amyotrophic lateral sclerosis (ALS), spinal cord injury, and other ailments have all been observed to benefit from it.

However, Li has a very narrow therapeutic window, and an overdose can cause tremors, urinary frequency, thyroid difficulties, being overweight, and renal failure. Clinical research of Li has revealed that an effective dosage range of 0.6–1.0 mM blood level (500–1200 mg Li per day) is beneficial, whereas hazardous levels occur at 1.2 mM or above. In 1985, the U.S. Environmental Protection Agency (EPA) suggested that a 70 kg adult consumes between 650 and 3100 mg of Li per day.

Previous evaluations of the Zn-Li system have demonstrated that the total amount of Li released from a Zn-Li stent (with 0.7 %wt Li) is twice less than the ideal daily intake; and the combination of Li and Zn would improve the UTS from 120 MPa (pure Zn) to 560 MPa (6 %wt Li). Owing to the greater volume fractions of the LiZn_4_ phase, an ample amount of Li meaningfully reduces ductility. The inclusion of Li should be kept below 4% to preserve appropriate ductility and strength for cardiovascular stent applications (0.4 %wt). Zhao et al. [67] created new Zn-Li alloy wires (with 0.1 wt% Li). Zn-0.1Li alloy wire implanted in the abdominal aorta of rats showed great biocompatibility in the arterial environment. When compared to Zn, Zn-0.1Li showed improved effective strengthening and cytocompatibility in implantations in orthopedics [83]. Yang et al. [84] completed a comprehensive study of biodegradable Zn alloys as orthopedic implants. Their findings showed that the alloys of Zn-Li, Zn-Mg, Zn-Ca, and Zn-Sr were the foremost required options for bone implants. Zn-Li alloys outperformed commercially pure Ti and stainless steel in terms of strength, indicating their potential for usage as high-load-bearing implants.

**Figure 5:**
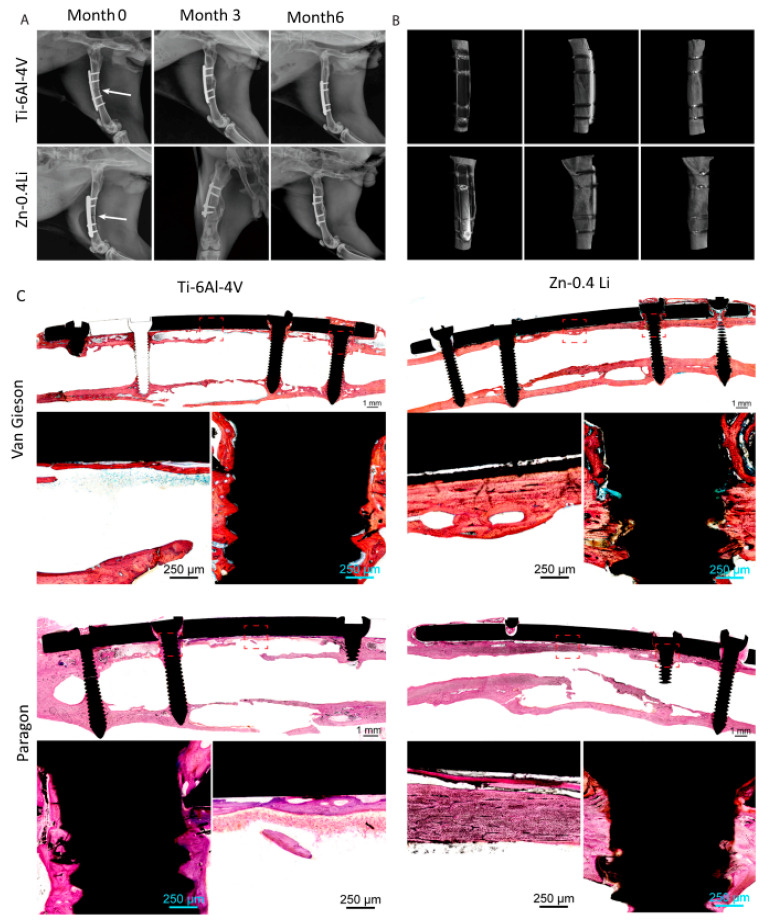
Zn-0.4Li fixation plate and screw in rabbit femoral shaft fracture model, with Ti-6Al-4 V alloy plate and screw as the control. (**A**) Representative X-ray images of rabbit femurs after 0, 3, and 6 months post surgery, fracture line is indicated by white arrows; (**B**) Micro-CT reconstruction of rabbit femoral shaft with plate and screw system at 6 months; (**C**) Van Gieson staining and Paragon staining of representative histological images of femoral fracture healing at 6 months. The fracture healing and fixation screws are magnified and marked by red rectangles. (Reproduced with permission from Ref. [84]. Copyright © 2021 Elsevier).

In the study, the authors integrated in vitro and in vivo tests to look into the osteogenic behavior and biological mechanism of Zn-Li alloys. They used a rabbit shaft fracture model with Ti-6Al-4 V alloy as an impact to check using the Zn-Li alloy as a load-bearing bone implant, its load-bearing capabilities, and bone fracture curing. X-ray scans taken after surgery revealed that the fracture ends were well aligned. Both implants showed signs of progressive healing over time (Figure 5A). After 3 months, the fracture line faded and was substituted by cortical bone bridging at the fracture place. 

In both groups, callus development was observed. The Ti-6Al-4 V alloy group had a larger callus tissue area than the Zn-0.4Li alloys. After 6 months, a micro-CT scan indicated that the bone fracture had been entirely cured and the fracture line had vanished (Figure 5B). Both sets remodeled their bone matrix and shape to be more typical. The tissue reaction during fracture healing was studied using Von Gieson and Paragon staining (Figure 5C). At six months, both groups had generated new cortical bone at the fracture location. The cortical bone in the Zn-0.4Li alloy group was substantially thicker than the Ti-6Al-4 V alloys, showing equivalent thickness to the proximal and distal normal cortical bone. In both groups, bone developed around the screw thread, indicating excellent osteointegration. It is worth noting that the Zn-0.4Li alloys had more osteocytes in their nascent bone tissue than the Ti-6Al-4 V alloy group. There was no evidence of gas production surrounding the Zn-0.4Li implants. Consequently, the Zn-0.4Li alloy performed as well as or better than the Ti-6Al-4 V alloy in the repair of bone fractures [84].

### 2.6. Zn-Ag Alloys

To boost the mechanical characteristics of Zn for stenting applications, the alloying of Zn is inevitable. As a result, the discovery of Zn alloys that may increase mechanical qualities while avoiding deleterious inflammatory reactions when compared to Zn and inert materials would be a significant step forward in the biodegradable implant sector. Owing to its capacity to increase tensile strength by grain refinement, as well as its acceptable biocompatibility and pronounced antibacterial characteristics, silver (Ag) has lately been proposed as a desirable alloying addition to Zn. Ag does, in fact, increase antibacterial activity in a variety of chemical states, allowing it to successfully destroy superbugs that are resistant to most antibiotics [85].

Several studies have investigated developing and manufacturing Zn alloys to improve their mechanical properties and make them more suitable for use as implants inside the human body. For instance, Li et al. [68] produced and evaluated a unique Zn-4%wtAg alloy as a possible BM in their research. In comparison to pure Zn, a thermomechanical treatment was used to enhance the microstructure and, as a result, boost the mechanical characteristics. The Zn-4 %wtAg alloy’s YS, UTS, and elongation are 157 MPa, 261 MPa, and 37%, respectively. This alloy has a corrosion rate of 0.75 ± 0.16 µg cm^2^ day1 determined from liberated Zn ions in DMEM extracts, which is greater than Zn. In vitro cytotoxicity experiments revealed that the Zn-4 %wtAg alloy is safe for L929 and Saos-2 cells, and that it can successfully prevent early bacterium adherence [68].

**Figure 6:**
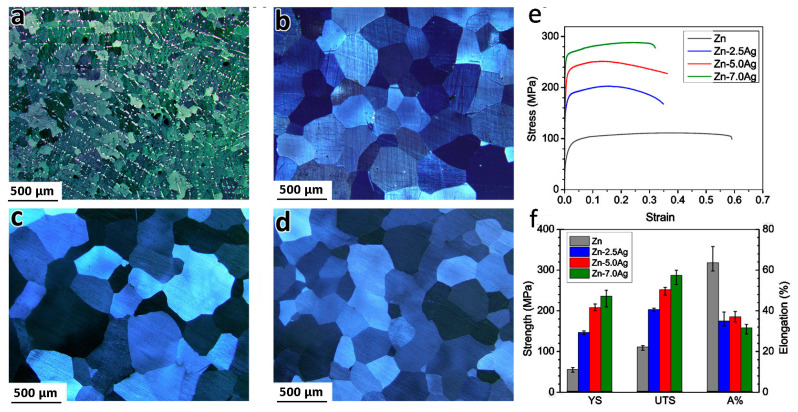
Microstructure of the solution-treated alloys at 410 °C for (**a**) Zn-7.0Ag after 6 h, (**b**) Zn-7.0Ag after 12 h, (**c**) Zn-2.5Ag, (**d**) Zn-5.0Ag after 6 h; room temperature tensile curves (**e**) and tensile properties of the extruded investigated materials (**f**). (Reproduced with permission from Ref. [69]. Copyright © 2017 Elsevier).

Sikora-Jasinska et al. [69] created Zn-Ag alloys with Ag content in the range of 2.5–7.0 %wt. The alloys were created using a casting technique and homogenized at 410 °C for 6 and 12 h, respectively, before being hot extruded at 250 °C with a 14:1 extrusion ratio. Hot extrusion greatly lowers the grain size of the alloys, according to microstructural studies. With a mean grain size of 1.5 µm, the Zn-7.0% Ag alloy has a very equiaxed and refined microstructure. The AgZn_3_ is broken into equal particles that are evenly distributed in the matrix with a reduced VF (Figure 6a). Figure 6b demonstrates that solution annealing for 12 h leads to the complete dissolution of the ε-AgZn_3_ phase in the η-Zn phase and forms a supersaturated solution of Ag in η-Zn. For 2.5% and 5.0% Ag alloys, the supersaturated solid solution can be gained after only 6 h, attributable to the lower silver concentration (Figure 6c,d). Tensile testing at ambient temperature revealed that gradually adding the Ag concentration improves tensile strength, while having no effect on tensile ductility (Figure 6e,f). Owing to grain refinement and a large VF of tiny AgZn3 particles precipitating at grain boundaries during the extrusion, Zn-%7.0 Ag has a high YS and UTS of 236 and 287 MPa, respectively. Zn-%7.0 Ag also exhibited superplasticity throughout a wide range of strain rates (from 5 × 10^−4^ s^−1^ to 1.0 × 10^−2^ s^−1^), allowing for their use in forming processes at high speeds and/or at low temperatures [69]. 

### 2.7. Zn-Cu Alloys

Copper (Cu) has too been utilized as an alloying component. Cu is a pivotal minor element for humans, since it impacts particular genes and acts as a co-factor or prosthetic for a number of enzymes. Cu levels in ordinary blood serum extend from 74 to 131 mol/L, with an everyday constraint of 3.0 mg per person. Additionally, Cu has been proven to advance endothelial cell multiplication and angiogenesis. As a result, when used as cardiovascular stents, Zn-based alloys comprising Cu may be capable of speeding up the endothelialization process. Moreover, Cu has an antibacterial action, which may help diminish the risk of infection in surgery. However, an abundance of Cu within the human body has been related to neurodegenerative sicknesses such as Alzheimer’s, Menkes, and Wilson’s disease [86].

**Figure 7:**
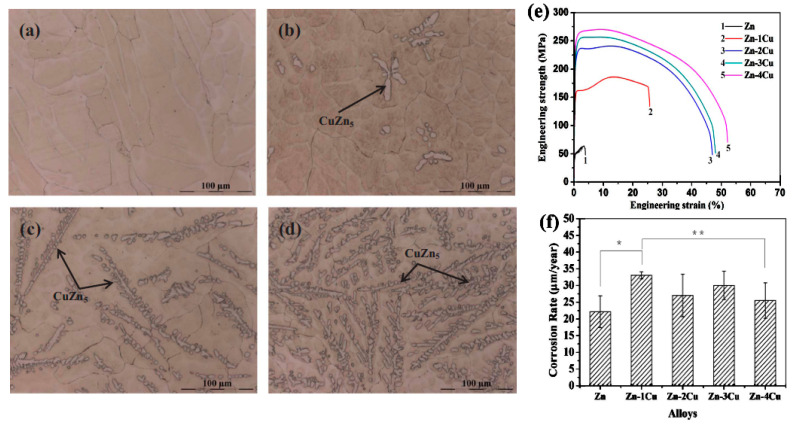
Microstructures of the as-cast Zn-xCu alloys. (**a**) Zn-1Cu; (**b**) Zn-2Cu; (**c**) Zn-3Cu; (**d**) Zn-4Cu; (**e**) Representative engineering stress vs. engineering strain curves of the as-extruded pure Zn and Zn-xCu (x = 1, 2, 3 and 4 wt%) alloys and (**f**) the corrosion rates of the as-extruded pure Zn and Zn-xCu alloys (x = 1, 2, 3 and 4 wt%) immersed in c-SBF solution for 480 h at 37 °C, * *p* < 0.05, ** *p* > 0.05. (Reproduced with permission from Ref. [70]. Copyright © 2017 Elsevier).

Tang et al. [70] developed a double Zn-Cu alloy as a BM to utilize in cardiovascular implants. Zn-Cu alloys are developed and manufactured using standard casting and hot extrusion. It has been indicated that when the Cu level rises, more CuZn5 phases form (Figure 7a–d). The CuZn_5_ phases are broken and the grains of the Zn-xCu alloys are refined after extrusion. Cu inclusion has helped to make strides in considerably advancing the mechanical features of Zn-xCu alloys through tensile tests (Figure 7e). The elongation of the Zn-4Cu reaches 50.6 ± 2.8%, which should ease the processing of micro-tubes for stent manufacturing. Zn-Cu alloy has been successfully used to produce micro-tubes with an external diameter of 3 mm and a thickness of 0.2 mm, as well as vascular stents. In c-SBF solution, the degradation rates of Zn-xCu alloys are relatively low, ranging from 22.1 ± 4.7 to 33.0 ± 1.0 µm year^−1^ (Figure 7f). The corrosion rates of Zn-xCu alloys expanded to some degree as the Cu content increased, when compared to Zn, which showed no substantial variation. In vitro tests have revealed that Zn-xCu alloys have an acceptable level of cytotoxicity to human endothelial cells, and that the antibacterial activity is perfect when the Cu content is more than 2 wt% [70].

Qu et al. [87] explored the antibacterial adequacy of Zn-Cu alloys utilizing efficient characterizations that combined in vitro and in vivo tests. The Zn-2Cu alloy had good mechanical qualities, as well as good biocompatibility and osteogenic features. Notably, the Zn-2Cu alloy inhibited bacterial adherence and biofilm development in both coagulase-positive and coagulase-negative staphylococci. In vivo, the Zn-2Cu alloy also prevented Methicillin-Resistant Staphylococcus Aureus (MRSA) infection and decreased its inflammatory toxic side effects. The Zn-2Cu alloy inhibited the expression of genes involved in wall synthesis, adhesion, colonization, biofilm arrangement, autolysis, and virulence factor secretion in MRSA, according to the PCR results. Therefore, biodegradable Zn-2Cu alloy is anticipated to be suitable for an assortment of antibacterial orthopedic implants [87].

### 2.8. Zn-Mn Alloys

It has been revealed that Mn promotes osteoblast proliferation, adhesion, and distribution, upregulates the expression of bone recovery marker genes such as alkaline phosphatase (ALP) and bone morphogenetic protein (BMP), advances collagen fiber regeneracy and deposition, and controls bone remodeling. It also aids in the preservation of bone mass [88]. Mn supplementation substantially inhibits bone loss in an ovariectomized rat model. In addition, Mn salt has insulin-like actions that can increase angiogenesis, speeding up the curing of femoral fractures in rats [89]. According to a recent study by Jia et al. [71], adding Mn to Zn greatly enhanced its elongation (elongation > 70%), demonstrating good deformability in the fabrication of implants with complex structural designs. Mn influences bone development at many sizes and levels in terms of bone metabolism. The resultant Zn-0.8 wt.% Mn alloy has an extraordinarily high elongation (83.96 ± 2.36%). Furthermore, cell proliferation and morphology tests have revealed that Zn-Mn alloys have much better cytocompatibility than Zn. Notably, the addition of Mn resulted in considerably enhanced osteogenic activity in terms of ALP activity and osteogenic expression. Micro-CT and histological results revealed boosted osteogenic activities for Zn-0.8Mn alloy in comparison to Ti, while H&E staining and blood tests confirmed the excellent in vivo biosafety of Zn-0.8Mn alloy. Zn-Mn alloys are reasonable candidate materials in bone defect treatment and fracture repair due to their excellent mechanical performance and excellent osteogenic ability [71,90]

Sun et al. [91] recently produced biodegradable Zn-Mn alloys with good overall characteristics, which they aim to use in therapeutic applications. At room temperature, double Zn-1%wt Mn alloys have exceptional ductility, which is beneficial for production and construction, and presents a lot of potential for strengthening. The inclusion of minor alloying components, particularly Cu and Ca, strengthens the Zn-0.8Mn alloy and increases its biocompatibility. MnZn_13_ is the most common second phase in Zn-Mn alloys, with a hardness of around 136 HV, greater than Zn. This phase is critical for increasing the alloys’ mechanical features [91].

Sotoudeh Bagha et al. [61] generated a biodegradable nanostructured alloy created using the mechanical alloying method. A powder metallurgy tactic was employed to synthesize nanostructured alloy from the milled powder. The mechanical features of Zn-4Mn showed higher compression strength and elongation at break than Zn-24Mn. MnZn13 is a second phase in alloys that influences mechanical and corrosion features. Zn, Zn-4Mn, and Zn-24Mn alloys had compressive yield strengths of 33, 290, and 132 MPa, respectively [61].

**Figure 8:**
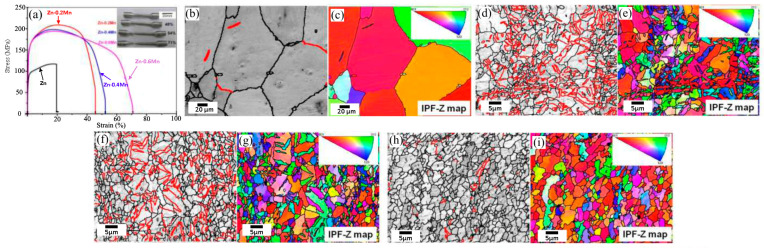
(**a**) Engineering stress-strain curves of extruded Zn-Mn alloys containing varying amounts of Mn and macroscopic images of specimens after tensile testing; EBSD maps of extruded Zn-Mn alloys with different Mn contents (**b**,**c**) Zn; (**d**,**e**) Zn-0.2Mn alloy; (**f**,**g**) Zn-0.4Mn alloy; (**h**,**i**) Zn-0.6Mn alloy; The {10–12} tensile twins are displayed in red (86.5° <11–20> ± 5°). (Reproduced with permission from Ref. [63] Copyright © 2017 Elsevier).

Sun et al.’s [63] study demonstrated that the volume fractions of tensile twins declined from 14.2 to 2.3% as the Mn content increased from 0.2 to 0.6 wt%. The number of MnZn13 compounds was also increased, though the tensile strength of the extruded Zn-Mn alloy diminished from 220 to 182 MPa and the elongation augmented from 48 to 71% (Figure 8a). Figure 8c–i represents the microstructure of the sample gained through Electron Backscatter Diffraction (EBSD).

Wide-ranging recrystallization followed by coaxial grain formation appears to occur in all extruded Zn-Mn alloys, and the Zn matrix grains are substantially refined by the addition of Mn (Figure 8). The average grain size of the extruded Zn-Mn alloy diminishes from 4 to 2 µm as the Mn content increases from 0.2 to 0.6 wt%, which is much smaller than pure extruded Zn (82 µm). In addition, tensile twins {10–12} are detected in the extruded Zn-Mn alloy. In Figure 8b,d,f,h, a tensile twin with an angle of rotation of 86.5° around a <11–20> axis is shown by a red line. The VF of the tensile twin in extruded Zn is 1.4% and the addition of 0.2 wt% Mn increases its value to 14.2%. As a result, as the manganese content increases, its VF declines. Furthermore, according to Figure 8c,e,g,i, most of the grains of all extruded Zn-Mn alloys have a (0001) orientation. This means that the texture does not depend on the Mn content. Therefore, tensile twins are an important item changing the mechanical features of extruded Zn-Mn alloys [63].

### 2.9. Zn-Mg-X (X = Sr, Ca, Mn, Fe, Cu, Al) Alloys

In the previous sections, the addition of Mg to Zn was addressed, and studies indicated that Mg can significantly enhance the hardness and tensile strength of Zn alloys. To modify the mechanical characteristics of Zn-Mg alloys, some elements, including Sr, Ca, Mn, Fe, Cu, and Al, are introduced [49].

#### 2.9.1. Zn-Mg-Sr\Ca Alloys

Ca and Sr are useful elements in the manufacturing of biological implants consisting of Zn alloys. They (Ca, Sr) are one of the main components of bone that are involved in mineralization, as well as being basic elements of chemical signals inside the cell and in enzymatic reactions in the human body. Liu et al. [49] added Ca and Sr to Zn-1Mg alloys, and demonstrated that these elements improved the hardness and mechanical strength of Zn. Then, they evaluated the addition of high levels of alloying elements, Ca 0.1 and Sr 0.1, to Zn-1.5Mg alloy and their effect on the mechanical and corrosion properties. This research showed that Zn-1.5 Mg alloy had a maximum UTS, a relatively low YS, and elongation (150.55 ± 13.57) MPa, (112.29 ± 2.99), MPa, (1.25 ± 0.16)%, respectively. After adding Ca, Sr casting elements, elongation, YS, and UTS increased to (2.03 ± 0.22)% (209.22 ± 9.96) MPa and (129.55 ± 7.57) MPa, for Zn-1.5Mg-0.1Sr alloy, and (1.72 ± 0.01)%, (173.81 ± 15.09) MPa, (241.38 ± 0.39) MPa, for Zn-1.5Mg-0.1Ca alloy, respectively [49].

**Figure 9:**
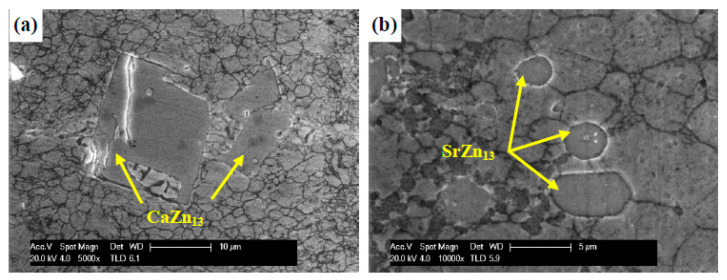
SEM images of (**a**) CaZn13 particles and (**b**) SrZn13 particles in 12p alloys. (Reproduced with permission from Ref. [92]. Copyright © 2020 Elsevier).

Huang et al. [92] added Ca and Sr to Zn-0.6% Mg alloy, (0.1 wt%) of Ca and Sr, and the ECAP (Equal Channel Angular Pressing) technique was used, which is a technique for severe plastic deformation (SPD) for the purpose of changing the shape of samples and preparing a uniform microstructure. This multi-pass ECAP method was used on Zn-Mg-Sr and Zn-Mg-Ca alloys. During the multi-pass ECAP process, it was concluded that the Zn-Mg-Sr and Zn-Mg-Ca alloys comprised of α-Zn matrix and network-shaped α-Zn + Mg_2_Zn_11_ + MgZn_2_ eutectic structure, and cuboid CaZn_13_ particles and ellipsoidal SrZn13 particles were obtained in the alloys, respectively (Figure 9). The results also displayed an increase in tensile strength after the multi-pass ECAP process, where the tensile strength improved to more than 300 MPa, and the ductility of the ECAP Zn-Mg-Sr alloy enhanced to more than 22%; in the Zn-Mg-Ca alloy, the ductility decreased to less than 10%. This is due to the influence of the cubic CaZn_13_ particles, which initiate initial failure through the formation of mold corners and increase stress concentration during deformation at the interface [92].

#### 2.9.2. Zn-Mg-Mn Alloys

Mn plays a major role inside the human body in activating multiple enzymatic including kinases, hydrolases, decarboxylases, transferases, and mitochondrial respiration without toxic effects. Mn can also greatly improve mechanical performance. Mn increases the corrosive ability of galvanic cells with higher electrode potentials, and thus has an impact on the corrosion properties by eliminating heavy metal components during the casting method. However, there have not been many systematic studies on Zn-, Mg-, and Mn-based alloys for biomedical applications [17,93].

Due to the benefits of Mn, Liu et al. [94] designed a new Zn-Mg alloy and added a small amount of Mn for the purpose of developing and improving the properties of the Zn-Mg-Mn alloy. The pure Zn presented very low YS and UTS, and a hardness of 22.85 MPa, 29.75 MPa, and 36.57 Hv. However, it was evident that after alloying with Mg and Mn, the YS, UTS, and hardness were altogether upgraded to 114.10 MPa, 131.94 MPa, and 97.66 Hv for Zn-1Mg-0.1Mn alloy; 114.71 MPa, 121.72 MPa, and 148.69 Hv for Zn-1.5Mg-0.1Mn alloy. Furthermore, the YS, UTS, and hardness of the Zn-1Mg-0.1Mn alloy after hot rolling were further improved (195.02 MPa, 299.04 MPa, and 107.82 Hv, respectively), and thermal deformation was recommended to boost its mechanical features. As for the results of the corrosion test, it was shown that Zn-1Mg-0.1Mn alloy had the best corrosion resistance at 0.11 mm/year after 30 days of immersion, and the electrochemical corrosion rate was 0.25. Furthermore, platelet adhesion and hemolysis rate results showed no evidence of thrombogenicity. The rate of hemolysis was low, with excellent blood compatibility [94,95].

#### 2.9.3. Zn-Mg-Fe Alloys

Shao et al. [96] demonstrated that the Zn-Mg-Fe alloy is nontoxic and has acceptable biodegradability properties. The Zn-Mg-Fe alloy osteosynthesis system was shown to be safe for usage within the body. Certain indicators are routinely used as clues in physiological and pathological conditions. These indicators are detected in regular blood and biochemical tests that correspond to numerous pathological changes in the body. Blood trace element indices are a good approach by which to track Zn levels in the blood and the Zn-Mg-Fe alloy’s degradation in vivo. The purpose of Shao’s group was to study the biocompatibility and degradability of a biodegradable Zn-Mg-Fe alloy osteosynthesis system. They noticed that several indicators varied within normal limits or displayed distinctive differences. However, there were no statistically significant changes, suggesting that there were no major blood cell abnormalities, renal dysfunction, hepatic, metabolism, lipid, or trace element problems throughout the alloy implantation. There is an osteosynthesis system in the frontal bone, mandible, and femur. The Zn concentration of bone rose for three months after implantation, and then declined, indicating that the Zn-Mg-Fe alloy was degrading. The Zn-Mg-Fe alloy uniformly disintegrated across the frontal bone, mandible, and femur implants, with no notable variations in degradation rates. In the first three months, the deterioration rate was around 0.183 mm y 1, and then dropped to around 0.065 mm y 1 after a year. Zinc oxide, zinc hydroxide [Zn(OH)_2_], hydrozincite [Zn_5_(OH)_6_(CO_3_)_2_], and hopeite [Zn_3_(PO_4_)_2_·4H_2_O] were the degradation products [96].

## 3. Zn-Based Composites

Pure Zn has weak mechanical characteristics, with a tensile strength of about 120 MPa and an elongation of about 2%, which is far from the strength required as a material for orthopedic implants (a tensile strength of 200 MPa or more and an elongation of 10% or more). Composite making is another practical method by which to boost the mechanical features of Zn and its alloys [45,97]. The deformation of materials with an HCP lattice, such as Zn alloys, at ambient temperatures is a challenge owing to the restricted number of slip systems. Zn alloys can be strengthened by incorporating reinforcement materials such as ceramic particles, carbon fibers, and metal particles to make Zn-based composites using a low-cost manufacturing method [39,98,99].

### 3.1. Carbon Elements Reinforcement

The implementation of nanotechnology in materials science creates new research and application opportunities for the fabrication of novel metal matrix nanocomposites (MMNCs). Nanomaterials with well-defined properties offer great prospects for use as bio-metal-reinforcing materials. Strengthening mechanisms in nano dimensions can be called Orowan’s formula and load transfer strengthening mechanisms, which have a great capacity to improve the mechanical behavior of metal matrix matrices. Carbonaceous materials such as the graphene family and carbon nanotubes (CNTs) are now emerging as major new materials for engineering applications as reinforcements for metallic matrix [6,7,100,101,102,103]. Considered one-dimensional (1D) structures, CNTs consist of rolled layers of carbon atoms. Their structure has a high elastic modulus of 1 TPa and their ultimate strength can reach 200 GPa. GNP, a novel nano-sized two-dimensional (2D) reinforcement with an ultra-high aspect ratio, consists of aggregated layers of nanocrystalline graphite. This reinforcement exhibits a breaking strength of up to 125 GPa and a modulus of elasticity of 1 TPa [101].

**Figure 10:**
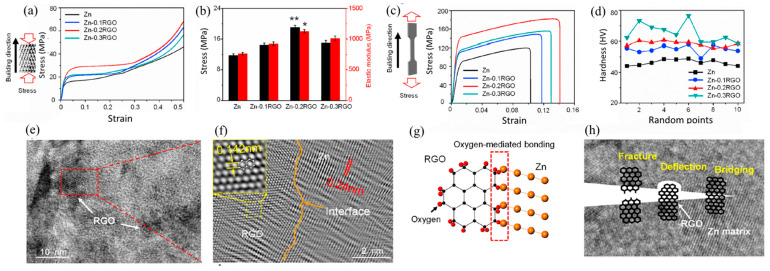
(**a**) Representative compressive curves of the Zn and Zn-RGO scaffolds; (**b**) The corresponding compression strength and elastic modulus; (**c**) Typical tensile curves of the Zn and Zn-RGO samples; (**d**) Vickers hardness. N = 3, * *p* < 0.05, ** *p* < 0.01 (Zn as control), (**e**,**f**) TEM images showing the interface bonding of RGO in the Zn matrix; (**g**) Schematic showing the oxygen-mediated bonding between Zn and RGO; (**h**) The possible strengthening mechanisms of RGO, which could effectively limit the crack propagation in the composite [104].

Reduced graphene oxide (RGO) was used as an additive for Zn-based scaffolds in a study by Youwen Yang et al. [104]. The experiments demonstrated that the uniformly dispersed RGO increased the strength and ductility of the Zn scaffold (Figure 10). In other words, the increase in strength was due to (1) grain refinement due to the pinning effect of RGO, (2) efficacious load transfer as a result of the large specific surface area of RGO and good interfacial bonding between RGO and Zn matrix, and (3) Orowan reinforcement with uniformly distributed RGO. The increased ductility was attributed to the random orientation of grains induced by RGO, which reduced the texture index from 20.5 to 7.3, activating more slip systems and providing more room for dislocation accommodation. 

**Figure 11:**
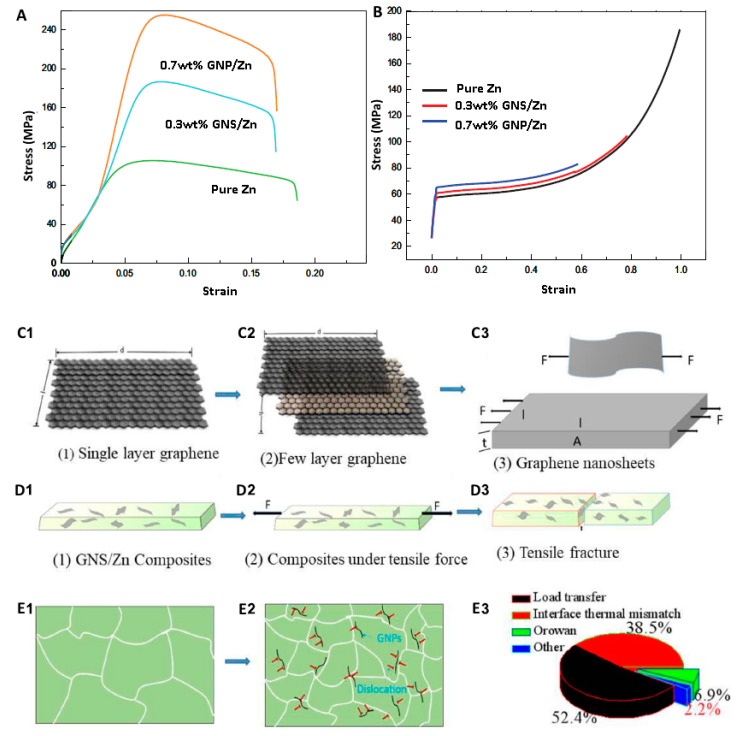
Pure Zn and GNS/Zn composites (**A**) tensile stress-strain curve, (**B**) compressive stress-strain curve. GNS/Zn composite tensile physical model (**C1**–**D3**), tissue evolution process (**E1**,**E2**), contribution diagram of strengthening mechanism (**E3**) [98].

Dai et al. [98] fabricated graphene nanosheets (GNSs) reinforced with Zn-based composites using spark plasma sintering (SPS). The experiments showed that the Zn/GNSs composites produced by SPS have a dense structure and good interfacial bonding, and the GNS are homogeneously distributed in the Zn matrix. The addition of 0.7 wt% GNS could remarkably enhance the mechanical behaviors of the composite (Figure 11). It has a UTS of 254 MPa and a Vickers microhardness of 65 HV, which are 126 and 20.3% higher than Zn (112 MPa and 54 HV, respectively). The strengthening mechanism of Zn/GNS composites is principally dislocation-strengthening, induced by coefficient of thermal expansion (CTE) inconsistency and GNS load transfer [98]. Fan et al. [105] produced Zn-based composites reinforced with partially unzipped MWCNTs (PUCNT) using spark plasma sintering (SPS). The strength of the PUCNT/Zn composites displayed a shift in terms of first increasing and then decreasing with increasing PUCNT content. At a 0.2 wt% PUCNT level, the UTS and YS of the composites were about 78.4% and 64.4%, respectively, which were higher than Zn while maintaining high elongation (62.6%). Table 3 shows the effect of adding reinforcements to the mechanical properties of Zn-based composite.

### 3.2. Bioceramic Reinforcements

The development of bioresorbable Zn matrix compounds using bioceramic reinforcements such as HA, TCP, MgO, etc., is a promising strategy by which to achieve higher mechanical activity and decent osseointegration as temporary orthopedic implants. Nevertheless, there have been few reports on Zn-based composites in the literature. 

Mahesh et al. [112] developed Zn-HA composites using the powder metallurgy method for use in temporary implants. They subjected Zn-HA powders with different amounts of HA (1, 2 and 4% by wt%) to ball milling for 1 h and then sintering. Microstructural observation revealed the formation of lamellar structures in the composite due to the plastic deformation of Zn powder during ball milling. The results showed that the particle size reduced with an increased loading of HA. Furthermore, measuring the granule’s length-to-thickness ratio showed that the aspect ratio decreased with increasing HA. The hardness value for all samples increased with the addition of reinforcement, and the highest hardness value was related to a 1 wt% reinforcement [112]. In another study, a Zn composite containing 8 wt% hydroxyapatite (Zn/HA8) was produced for the first time by extrusion. The extrusion process resulted in good interfacial integrity between Zn and HA particles. The recrystallization of the Zn matrix during extrusion was promoted near the HA particles. The mechanical behavior confirmed the role of HA as a material structural defect; the mechanical properties of Zn/HA8 decreased by about 30% (CYS = 154 MPa Zn, 113 MPa Zn /HA8). Nevertheless, the Zn/HA8 composite provided sufficient mechanical activity to replace cancellous bone and reach the range of cortical bone [108]. Narendra Kumar [113] and colleagues made Zn- magnesium oxide (MgO) composites (0, 1, 2, and 3% MgO) using a powder metallurgy method. The grain size slightly decreased with increasing MgO percentage in the composite due to the addition of MgO, which reduced grain growth during sintering. The highest hardness value belonged to the Zn-2% MgO sample [113]. Beta-tricalcium phosphate (β-TCP) is one of the most investigated biomedical reinforcers. As a bioactive ceramic, β-TCP is biocompatible, osteoconductive, and biodegradable. Adding β-TCP not only significantly boosts the mechanical features of the material, but also the biocompatibility of Mg alloys [110]. Tensile tests showed that the Zn-1Mg-1vol%β-TCP composites had optimum mechanical characteristics. The YS, UTS, elongation (σ), and Young’s modulus (E) of the as-extruded Zn-1Mg-1vol%β-TCP composite is 250.8 MPa, 330.5 MPa, 11.7%, and 125.4 Gpa, respectively. Moreover, β-TCP enhances the cytocompatibility of the composite over the Zn-1Mg alloy [110].

**Figure 12:**
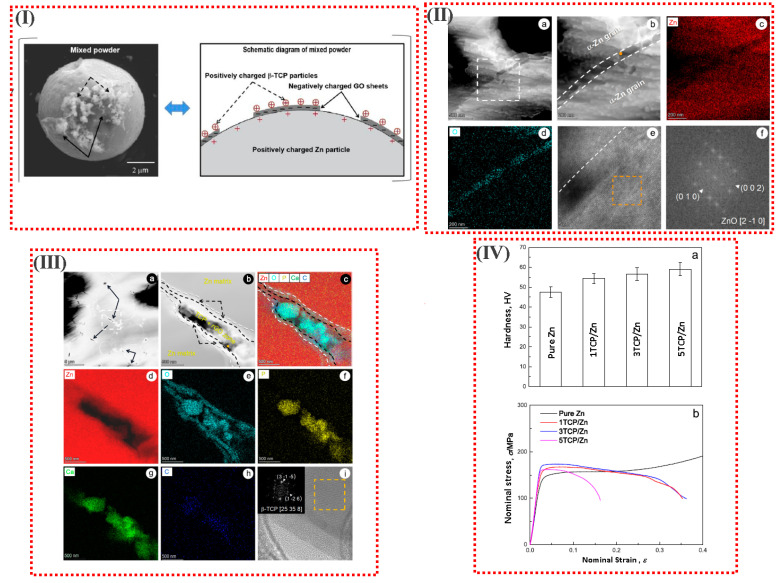
(**I**) Schematic diagram of the self-developed GO-assisted hetero-agglomeration method, the typical SEM images and schematic of the mixed powder prepared via the self-developed procedures. (**II**) (**a**,**b**) high-angle annular dark-field imaging (HAADF) images and (**II**) (**c**,**d**) TEM-EDS element mapping results of the pure Zn components fabricated using the SPS process; (**II**) (**e**) high resolution (HR)-TEM image observed from orange solid circle area inserted in (**II**) (**b**); (**II**) (**f**) the fast Fourier transform (FFT) pattern of crystalline ZnO taken from orange dashed square inserted in (**II**) (**b**). (White dashed frame inserted in (**II**) (**a**) indicates the area of interest in (**II**) (**b**); white dashed lines indicate the boundary between the α-Zn grain and the nanoscale ZnO layer). (**III**) (**a**,**b**) high-angle annular dark-field imaging (HAADF) images and (**III**) (**c**–**h**) TEM-EDS element mapping results of the 3TCP/Zn composites; (**III**) (**i**) HR-TEM image observed from orange solid circle area inserted in (**III**) (**b**). (Black solid arrows refer to the TCP nanoparticles; white dashed frame inserted in (**IV**) (**a**) indicates the observation area of (**III**) (**b**); black dashed arrows indicate the two ZnO layers enveloping the TCP + rGO zone; white dashed lines indicate the boundary between the Zn grains and ZnO double layer; black dashed lines indicate the boundary between the ZnO double layer and the TCP + rGO zone; the FFT pattern of β-TCP taken from the orange dashed square inserted in the top left part of (**III**) (**i**)). (**IV**) (**a**) Vickers hardness and (**IV**) (**b**) compressive nominal stress-nominal strain curves of pure Zn components and TCP/Zn composites with different TCP contents [111].

In another study, Zn-based composites reinforced with β-TCP were uniformly produced through GO-assisted heterogeneous aggregation and SPS. A very low portion (0.04 vol%) of multilayer GO was utilized as a coupling agent to attach the Zn matrix and the nanoscale TCP particles. In suitable polar solvents, the negatively charged GO layer could associate with both the positively charged Zn powder and TCP particles due to electrostatic attraction and charge neutralization. Owing to hetero agglomeration, the flexible GO film adheres to the large Zn powder and attracts TCP particles to make a Zn/GO/TCP sandwich configuration due to charge neutralization and the TCP formed in it. After the SPS process, TCP particles embedded in a very thin ZnO layer (tens of nanometers thick) formed a uniform 3D network-like distribution in the TCP/Zn sintered composites. A “snap-pea”-like structure was approved at the grain boundary of the α-Zn grains, with the TCP grains as the “pea” and the thin ZnO film as the “pod”. As a result of the monotonous distribution of TCP bioactive particles and the unique grain boundary structure of TCP, the sintering YS of the 3TCP/Zn matrix composite was 140.8 ± 7.7 MPa and the elongation at break was 36.0 ± 2.8% (Figure 12) [111].

## 4. Manufacture Methods for Biodegradable Zn

### 4.1. Casting

Casting is the foremost process for manufacturing Zn-based alloys in terms of the straightforward alteration of the alloy composition. Heating metal components above their melting point (typically between 470 and 750 °C) in a vacuum or under Ar, SF_6_, or CO_2_ atmosphere, pouring the molten metal into suitable molds (e.g., steel or graphite), and permitting consolidation are all part of the procedure. Resistance and induction melting furnaces are the foremost common types of melting furnaces [114,115,116,117].

Most of the research indicates the use of Zn as a biomedical implant, so the metal should be shaped like a service form. Bone fixation methods, for example, require flat plates, while stents need thin-walled cylindrical tubes. Casting is not the best way to create mesh shapes. Cast metal, on the other hand, can still be used as the starting material for the subsequent molding process [4].

### 4.2. Conventional Wrought Procedures

**Figure 13:**
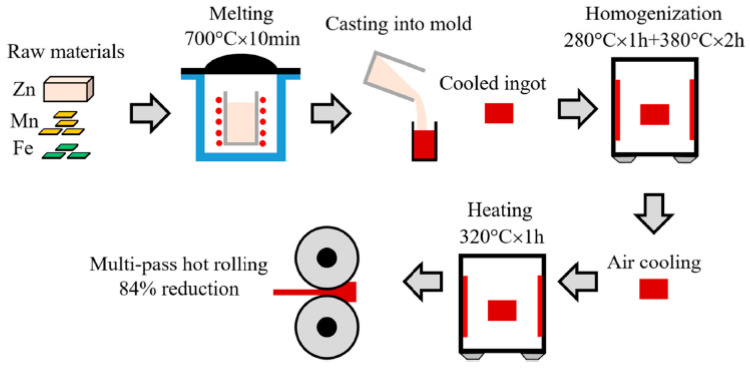
Fabrication schematic of Zn-based alloy plates by hot rolling [118].

Wrought procedures in metalworking describe the operation of plastically deforming metal into a desired shape using mechanical force. The word wrought implies ‘worked’, inferring that the process is mechanical. The wrought process can be hot or cold depending on the relationship between the working temperature and the recrystallization temperature of the metal. Hot working refers to the process of plastically shaping metal above its recrystallization temperature. Otherwise, it is cold working. Rolling, forging, extrusion, and drawing are the most common steps. Hot rolling is the prime technique for creating a flat profile in most studies of biodegradable Zn. Hot rolling is the process of reducing the thickness of a heated metal stock by passing it between a pair of rolls. In order to obtain a uniform composition within the preformed Zn, rolling is periodically performed before the homogenization treatment. This involves heating the metal at 250–350 Co for 30–180 min. The rolled product’s thickness might range from a few millimeters to roughly 300 mm. A schematic diagram of the fabrication process by hot rolling is provided in Figure 13 [118,119,120,121].

Extrusion or drawing can be used to construct the tube’s extended cylindrical profile. Extrusion is the process of forcing metal through a die with a specified form opening. Extrusion can be carried out in two ways: directly or indirectly. The die is stationary in direct extrusion, and the billet is forced through the die using a ram. The die is mobile in indirect extrusion, and it is pushed into the billet, causing the metal to flow through the opening. Drawing is identical to extrusion, except that instead of being pushed through the die, the metal is drawn through it. Before shaping, the metal is frequently preheated at 180–300 °C for 30–180 min in both methods. Extrusion is also commonly employed as an initial forming process before drawing for further reduction. Hollow tubes can also be made via extrusion or drawing, with the help of suitable mandrels. Hollow sinking and mandrel drawing were utilized by Wang et al. to manufacture microtubes with a diameter of 2.5 mm and a thickness of 130 mm. Mandrel drawing includes drawing the rolled tube through the space between the mandrel and the die, whereas hollow sinking requires drawing the tube via a drawing die [4,59,66,122,123,124]. 

### 4.3. Advanced Processing Methods

There have been a few papers published on the use of innovative production processes to make biodegradable Zn. Vida et al. [125] created cast Zn with intriguing microstructures using an improved solidification process termed directed solidification casting. The method is a variation of classic casting that employs a water-cooled mold that promotes directed solidification by manipulating heat transfer [125].

Jarzebska et al. [126] used cold hydrostatic extrusion, a kind of extrusion in which the billet is confined in a pressured liquid to manufacture 5 mm diameter Zn rods. The liquid pressure was kept between 470 and 500 MPa, while the strain rate was controlled between 4 and 40 s per second [126].

Dambatta et al. [127] employed ECAP, which is a kind of severe plastic deformation technique in which a metal billet is forced through an angled (e.g., 90) channel. They employed dies with a cross-section of 8 mm and a 120 C intersection angle; 1 mm s1 was the strain rate. To minimize friction loads, the dies were heated to 200 °C and lubricated with molybdenum disulfide [127].

Biodegradable Zn has also been made using powder metallurgy (P/M) processes [128]. S. Bagha et al. [61] used mechanical alloying on matching powders, followed by uniaxial cold pressing and sintering, to create dense Zn-Mn disks. The milling operation lasted 20 h in an argon environment with a 20:1 ball-to-powder weight ratio. The cold squeezing of the alloy powders was carried out at 300 MPa, and sintering was carried out for 1 h at 250 and 450 °C.

**Figure 14:**
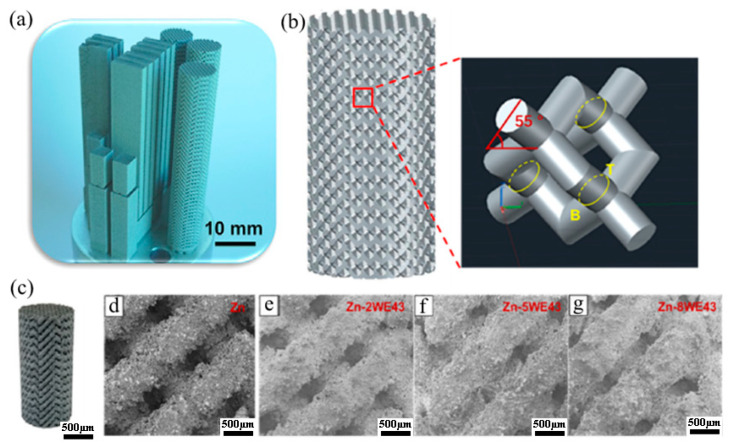
(**a**) Additively manufactured Zn-Xwe43 scaffolds made by laser powder bed fusion (L-PBF): (**b**) shape of porous scaffolds, (**c**) macro image, (**d**–**g**) enlarged parts of Zn-xWE43. (Reproduced with permission from Ref. [129]. Copyright © 2019. Elsevier).

Additive manufacturing can not only effectively and precisely attain macrostructures with a customized form, but can also deliver microstructures such as interconnected micropores or the diverse distribution of materials by specifically melting/bonding separate materials layer by layer with accuracy through computer monitoring (Figure 14) [129,130,131,132,133,134,135]. Porous Zn manufacturing is especially appealing because this material could be beneficial in various biological applications, such as the production of bioresorbable, tissue-engineered bone scaffolds, and additive manufacturing can be considered a perfect strategy for tissue engineering and bio-fabrication [136,137,138,139,140,141,142,143,144,145,146,147].

## 5. Conclusions and Future Works

Zn has been recognized as one of the most promising BMs. When used in biomedical implant applications, given that both metals of Mg and Fe continue to be a source of concern, scientists are searching for novel alternatives. Some of the fundamental obstacles to the use of biodegradable Mg and Fe appear to be overcome by Zn and its composites, and there has been increasing research on Zn metal in recent years. This paper reviews the current state-of-the-art on biodegradable Zn, including current advances, existing opportunities, and future research goals. The review focuses on the analysis of the microstructure, the mechanical and biological properties of Zn-based alloys and composites, as well as the alloying and fabrication processes. Zn demonstrates excellent corrosion rates and is suitable for bioresorbable implants. However, its poor mechanical properties have limited its medical applications. Adding alloying elements and reinforcements to Zn can have an important effect on improving its mechanical behavior. Although, few studies have been published in this field. It seems that no comprehensive research has been conducted on alloys and composites. Therefore, future research should include systematic and long-term comparisons and reviews to clearly identify the strengths and weaknesses of each composite.

## Figures and Tables

**Table 1:** Comparison of the mechanical properties and degradation rates of bone tissues along with existing non-biodegradable and biodegradable metallic materials.

Material Type	Density (g/cm^3^)	UCS (MPa)	UTS (MPa)	E (GPa)	ε (%)	Degradation Rate (mm y^−1^)	Refs.
Natural bone	1.8–2.1	164–200	35–283	3–20	3–4	NBR	[40,41]
Ti alloy	4.4–4.5	900	900–1000	110–127	10–15	No	[40,42]
Stainless Steel	7.9–8.1	500–1000	460–1700	189–205	10–40	No	[43]
Co-Cr alloy	7.9–8.1	450–1000	860	230	20	No	[39]
Mg pure	1.74–2	65–100	135–285	41–45	7–40	0.8–2.7	[40,44]
Zn pure	7.1	30–100	100–150	78–121	0.3–2	0.1–0.3	[45,46,47]
Fe pure	7.8	560	300	213	37.5	0.1	[11,48]

UCS: ultimate compressive strength; UTS: ultimate tensile strength; E: Young’s modulus; ε: elongation; NBR: natural bone remodeling.

**Table 2:** Effect of different elements on the mechanical properties of Zn-based alloys.

Mechanical Properties								
Tissue/Alloy	Processing Technique	Tensile Yield Strength (TYS) (MPa)	Ultimate Tensile Strength (UTS) (MPa)	Compressive Yield Strength (CYS)	Ultimate Compressive Strength (UCS)	Elongation (%)	Hardness (HV)	Ref.
Pure Zn								
Zn	Cast + HE	68	135	-	-	61.2	32	[58]
Zn	Cast	9.3	18.1	-	-	0.34	37.8	[46]
Zn	Rolled	30	50	-	-	5.62	40	[46]
Zn	Extruded	34.6	63.9	103.1	-	3.57	-	[46]
Zn	Cast	10 ± 2	18 ± 3	102.92 ± 6.73	-	0.3 ± 0.1	38.2 ± 1.0	[59]
Zn	HE	51 ± 3.7	111 ± 4.5	-	-	60 ± 5.9	34 ± 1.7	[60]
Zn	PM	-	-	-	33	16	18	[61]
Zn	HE	55 ± 8	97 ± 10	94 ± 13	-	77 ± 2.7	44 ± 6	[62]
Zn		60	117	-	-	14	-	[63]
Zn	HE	124	164	94	276	39.3	44	[64]
Zn-Mg								
Zn-1.5 Mg	Cast + HE	356	463	-	-	38.6	120	[58]
Zn-0.5 Mg	Cast + HE	373	514	-	-	10.5	107	
Zn-1Mg	Cast + HE	367	478	-	-	24.9	111	
Zn-1Mg	Cast	130 ± 10	180 ± 21	284.50 ± 16.90	-	2 ± 0.2	78.26 ± 2.84	[59]
Zn-0.15Mg	HE	114 ± 7.7	250 ± 9.2	-	-	22 ± 4	52 ± 4.9	[60]
Zn-0.5Mg	HE	159 ± 8.9	297 ± 6.9	-	-	13 ± 0.9	65 ± 3.9	[60]
Zn-1Mg	HE	180 ± 7.3	340 ± 15.6	-	-	6 ± 1.1	75 ± 3.9	[60]
Zn-3Mg	HE	291 ± 9.3	399 ± 14.4	-	-	1 ± 0.1	117 ± 7.1	[60]
Zn-0.8Mg	HE	203 ± 7	301 ± 8	186 ± 10	-	13 ± 2	83 ± 5	[62]
Zn-1.6Mg	HE	232 ± 8	368 ± 8	257 ± 13	-	4 ± 0.3	97 ± 4	[62]
Zn-1 Mg	Cast	-	153	-	-	1.5	65	[65]
Zn-0.1 Mg	HE	214	274	201	534	10.2	70	[64]
Zn-0.4Mg	HE	284	353	281	604	15.2	82	[64]
Zn-0.8 Mg	HE	297	386	304	631	9.3	96	[64]
Zn-Ca								
Zn-1Ca	Cast	119 ± 7	165 ± 14	280.7 ± 20.7	-	2.1 ± 0.2	73 ± 7.43	[59]
Zn-0.1	HE	127	169	122	289	37.9	45	[64]
Zn-0.4	HE	116	166	111	269	26.7	44	[64]
Zn-0.8	HE	127	173	111	303	27.9	44	[64]
Zn-Sr								
Zn-1Sr	Cast	120 ± 6	171 ± 14	340.9 ± 17.7	-	2 ± 0.2	61.8 ± 6.7	[59]
Zn-0.1Sr	HE	89	139	88	250	34.5	44	[64]
Zn-0.4Sr	HE	106	153	94	293	20.2	44	[64]
Zn-0.8Sr	HE	104	151	105	306	30.0	48	[64]
Zn-Al								
ZnAl4Cu1	Cast	171	210	-	-	1	80	[66]
Zn-0.5Al	As cast-Extruded	119 ± 2.3	2.3 ± 9.6	-	-	33 ± 1.2	59 ± 5.8	[60]
Zn-1Al	As cast-Extruded	134 ± 5.8	223 ± 4.3	-	-	24 ± 4.2	73 ± 4.6	[60]
Zn-Mg-Ca								
Zn-1Mg-1Ca	Cast	79.8	129.6	-	-	1.02	91.5	[46]
Zn-1Mg-1Ca	Rolled	127.1	197.1	-	-	8.37	106.7	[46]
Zn-1Mg-1Ca	Extruded	205.9	257.4	299.4	3272.4	5.35	-	[46]
Zn-Mg-Sr								
Zn-1Mg-1Sr	Cast	85.6	136.1	-	-	1.20	85.2	[46]
Zn-1Mg-1Sr	Rolled	139.2	202.1	-	-	9.63	91.8	[46]
Zn-1Mg-1Sr	Extruded	202.4	253.8	383.5	3848.3	7.44	-	[46]
Zn-Ca-Sr								
Zn-1Ca-1Sr	Cast	84.4	139.6	-	-	1.13	90.1	[46]
Zn-1Ca-1Sr	Rolled	142.8	202.8	-	-	8.73	86	[46]
Zn-1Ca-1Sr	Extruded	213.0	260.9	340.9	3244.3	6.76	-	[46]
Zn-Li								
Zn-Li	Cast	238 ± 60	274 ± 61			17 ± 7	97 ± 2	[67]
Zn-0.1Li	HE	341	431	306	784	28.1	108	[64]
Zn-0.4Li	HE	387	520	434	794	5	164	[64]
Zn-0.8Li	HE	-	-	454	1022	-	216	[64]
Zn-Ag								
Zn-4Ag	TT	157	261	-	-	37	73	[68]
Zn-4Ag	APH	149	215	-	-	24	82	[68]
Zn-2.5Ag		160	200	-	-	35	-	[69]
Zn-5.0Ag		210	260	-	-	39	-	[69]
Zn-7.0Ag		230	280	-	-	32	-	[69]
Zn-0.4Ag	HE	127	167	88	162	38.1	50	[64]
Zn-0.8Ag	HE	134	184	82	177	58.3	58	[64]
Zn-2.5Ag	HE	186	231	145	221	36.7	55	[64]
Zn-Cu								
Zn-1Cu	As cast- Extruded	148.7 ± 0.5	186.3 ± 0.5	-	-	21.0 ± 4.4	-	[70]
Zn-2Cu	As cast- Extruded	199.7 ± 4.2	240.0 ± 1.4	-	-	46.8 ± 1.4	-	[70]
Zn-3Cu	As cast- Extruded	213.7 ± 1	257.0 ± 0.81	-	-	47.2 ± 1	-	[70]
Zn-4Cu	As cast- Extruded	227 ± 5	270.7 ± 0.5	-	-	50.6 ± 2.8	-	[70]
Zn-0.4Cu	HE	150	197	139	451	40.2	59	[64]
Zn-0.8Cu	HE	184	234	165	495	33.1	69	[64]
Zn-2Cu	HE	223	270	233	527	40.7	75	[64]
Zn-Mn								
Zn-0.1 Mn	Extruded	125	175	-	-	40	-	[71]
Zn-0.4 Mn	Extruded	165	220	-	-	45	-	[71]
Zn-0.8 Mn	Extruded	165	175	-	-	85	-	[71]
Zn-4 Mn	PM	-	-	-	290.8	14.9	102	[61]
Zn-24Mn	PM	-	-	-	132.4	6.7	71	[61]
Zn-0.2Mn	As cast- Extruded	132	220	-	-	48	-	[63]
Zn-0.4Mn	As cast- Extruded	123	198	-	-	54	-	[63]
Zn-0.6Mn	As cast- Extruded	118	182	-	-	71	-	[63]
Zn-0.1Mn	HE	131	177	125	383	39.8	54	[64]
Zn-0.4Mn	HE	160	214	136	439	43.4	57	[64]
Zn-0.8Mn	HE	156	190	145	383	83.8	50	[64]

TT: thermomechanical treatment; APH: additional precipitation hardening.

**Table 3:** Effect of different reinforcements on the mechanical properties of Zn-based composites.

Mechanical Properties of Zn Based Composites								
Composite/Zn Based	Processing Technique	Tensile Yield Strength (TYS) (MPa)	Ultimate Tensile Strength (UTS) (MPa)	Compressive Yield Strength (CYS)	Ultimate Compressive Strength (UCS)	Elongation (%)	Hardness (HV)	Ref.
Carbon elements reinforcements								
Zn	LPBF	91.6 ± 7.3	119.9 ± 8.5	-	-	9.5 ± 10	-	[104]
Zn-0.1RGO	LPBF	111.3 ± 9.1	148.5 ± 10.6	-	-	11.7 ± 1.2	-	[104]
Zn-0.2RGO	LPBF	142.9 ± 13.4	182.1 ± 15.4	-	-	14.1 ± 1.8	-	[104]
Zn-0.3RGO	LPBF	115.7 ± 17.5	155.2 ± 18.1	-	-	12.9 ± 2.3	-	[104]
0.1PUCNTs/Zn	SPS	157	185	-	-	113		[105]
0.2PUCNTs/Zn	SPS	169	210	-	-	67	-	[105]
0.3PUCNTs/Zn	SPS	118	131	-		58	-	[105]
0.5PUCNTs/Zn	SPS	125	133	-	-	52	-	[105]
Pure Zn	SPS	-	112	-	77	-	54 (AF)	[98]
0.3GNS/Zn	SPS	-	187	-	100	-	58 (AF)	[98]
0.7GNS/Zn	SPS	-	254	-	185	-	65 (AF)	[98]
Pure Zn	M-ECD + PM	-	-	97	-	-	57	[106]
Zn/*f*-GNP	M-ECD-PM	-	-	182–284	-	-	68.8–108.5	[106]
Bioceramic reinforcements								
Zn	SPS	-	-	≈ 51	≈172	-	≈44	[107]
Zn-1HA	SPS	-	-	≈66	≈157	-	≈46	[107]
Zn-5HA	SPS	-	-	≈42	≈108	-	≈44	[107]
Zn-10HA	SPS	-	-	≈44	≈71	-	≈45	[107]
Zn	Ex	-	-	153.6 ± 11.1	243.8 ± 1.5	-	45.6 ± 2	[108]
Zn/8HA	Ex	-	-	112.8 ± 5.1	168.9 ± 3.7	-	44.7 ± 4.5	[108]
Zn	SPS	-	-	92.1 ± 1.2	128.7 ± 1.7	-	36.8 ± 1.4	[109]
Zn/HA8	SPS	-	-	67.9 ± 7.4	88.9 ± 7.2	-	34.3 ± 4.5	[109]
Zn-1Mg	UCHET	235.7 ± 5	315.2 ± 8	-	-	6.7 ± 2	-	[110]
Zn-1Mg-1TCP	UCHET	250.8 ± 7	330.5 ± 9	-	-	11.7 ± 3	-	[110]
Zn-1Mg-3TCP	UCHET	220.3 ± 8	308.1 ± 7	-	-	4.5 ± 2	-	[110]
Zn-1Mg-5TCP	UCHET	194.7 ± 8	299.4 ± 9	-	-	3.9 ± 3	-	[110]
Pure Zn	GHA-SPS	-	-	121.3 ± 6.8	156.9 ± 4.8	-	47.3 ± 2.7	[111]
1TCP/Zn	GHA-SPS	-	-	132.3 ± 5.3	167.5 ± 3.9	-	54.4 ± 2.5	[111]
3TCP/Zn	GHA-SPS	-	-	141.8 ± 7.7	173.9 ± 4.9	-	56.5 ± 3.2	[111]
5TCP/Zn	GHA-SPS	-	-	142.4 ± 6.4	161.8 ± 4.4	-	59.0 ± 3.3	[111]

AF: after rolling; GNS: graphene nanosheets; LPBF: laser powder bed fusion; f-GNP: functionalized graphene nano-platelet; Ex: extrusion; UCHET: ultrasonic-assisted casting and hot extrusion technology; TCP: beta-tricalcium phosphate; GHA: graphene oxide-assisted hetero-agglomeration.

## Data Availability

All data provided in the present manuscript are available to whom it may concern.
